# 1949. Activity of Aztreonam-Avibactam against Enterobacterales Resistant to Recently Approved Beta-Lactamase Inhibitor Combinations Collected Worldwide (ex-US; 2020–2022)

**DOI:** 10.1093/ofid/ofad500.103

**Published:** 2023-11-27

**Authors:** Helio S Sader, Mariana Castanheira, Leonard R Duncan, John H Kimbrough, Cecilia G Carvalhaes, Rodrigo E Mendes

**Affiliations:** JMI Laboratories, North Liberty, Iowa; JMI Laboratories, North Liberty, Iowa; JMI Laboratories, North Liberty, Iowa; JMI Laboratories, North Liberty, Iowa; JMI Laboratories, North Liberty, Iowa; JMI Laboratories, North Liberty, Iowa

## Abstract

**Background:**

Aztreonam-avibactam (ATM-AVI) is under clinical development. Aztreonam is a monobactam stable to hydrolysis by metallo-β-lactamases (MBLs). ATM-AVI has shown activity against MBL-producing, carbapenem-resistant Enterobacterales (CRE) that are resistant to recently approved β-lactamase inhibitor combinations (BLIs) such as ceftazidime-avibactam (CAZ-AVI), meropenem-vaborbactam (MEM-VAB), and imipenem-relebactam (IMI-REL). We evaluated a large collection of CRE isolates nonsusceptible (NS) to these BLIs.

**Figure:**
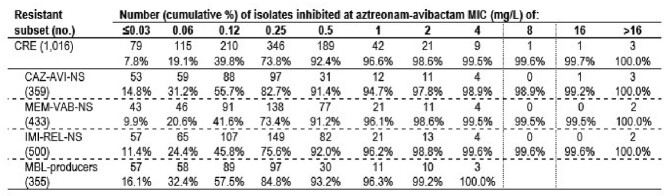

**Methods:**

24,580 Enterobacterales isolates were consecutively collected (1/patient) in 2020–2022 from 64 medical centers located in Western Europe (W-EU; 27 centers in 10 countries), Eastern Europe (E-EU; 13 centers in 9 countries), Latin America (LATMA; 8 centers in 6 countries), and the Asia-Pacific region (APAC; 16 centers in 8 countries). Among these isolates, 1,016 (4.1%) were CRE. Isolates were susceptibility tested by CLSI broth microdilution. CRE isolates were screened for carbapenemase (CPE) genes by whole genome sequencing.

**Results:**

ATM-AVI inhibited 99.6% of CREs (MIC_50/90_, 0.25/0.5 mg/L) and ≥ 98.9% of CRE isolates NS to CAZ-AVI, MEM-VAB, and/or IMI-REL at ≤8 mg/L (Table). The most active comparators against CREs were CAZ-AVI (64.6%S), MEM-VAB (57.4%S), and IMI-REL (50.7%S). The activity of these BLIs varied widely among region, with highest susceptibility rates observed in W-EU (76.9% for CAZ-AVI, 72.5% for MEM-VAB, 63.8% for IMI-REL), followed by LATAM (65.1% for CAZ-AVI, 70.6% for MEM-VAB, 62.8% for IMI-REL), E-EU (66.6% for CAZ-AVI, 46.7% for MEM-VAB, 43.5% for IMI-REL), and APAC (39.9% for CAZ-AVI, 37.8% for MEM-VAB, 27.7% for IMI-REL). The most common CPE types overall were KPC (44.5% of CREs), NDM (29.8%), and OXA-48-like (16.0%). KPC predominated in LATAM (64.1%) and W-EU (61.1% of CREs). MBL occurrence was highest in APAC (59.5% of CREs), followed by LATAM (34.0%), E-EU (28.9%), and W-EU (23.6%). NDM represented 85.4% of MBLs.

**Conclusion:**

ATM-AVI demonstrated potent activity against CRE isolates resistant to CAZ-AVI, MEM-VAB, and/or IMI-REL independent of the CPE produced. The activity of recently approved BLIs varied broadly among regions and were very limited in E-EU and APAC.

**Disclosures:**

**Helio S. Sader, MD, PhD, FIDSA**, AbbVie: Grant/Research Support|Basilea: Grant/Research Support|Cipla: Grant/Research Support|Paratek: Grant/Research Support|Pfizer: Grant/Research Support|Shionogi: Grant/Research Support **Mariana Castanheira, PhD**, AbbVie: Grant/Research Support|Basilea: Grant/Research Support|bioMerieux: Grant/Research Support|Cipla: Grant/Research Support|CorMedix: Grant/Research Support|Entasis: Grant/Research Support|Melinta: Grant/Research Support|Paratek: Grant/Research Support|Pfizer: Grant/Research Support|Shionogi: Grant/Research Support **Leonard R. Duncan, PhD**, AbbVie: Grant/Research Support|Basilea: Grant/Research Support|CorMedix: Grant/Research Support|Melinta: Grant/Research Support|Pfizer: Grant/Research Support **John H. Kimbrough, PhD**, AbbVie: Grant/Research Support|Basilea: Grant/Research Support|Pfizer: Grant/Research Support|Shionogi: Grant/Research Support **Cecilia G. Carvalhaes, MD, PhD**, AbbVie: Grant/Research Support|bioMerieux: Grant/Research Support|Cipla: Grant/Research Support|CorMedix: Grant/Research Support|Melinta: Grant/Research Support|Pfizer: Grant/Research Support **Rodrigo E. Mendes, PhD**, AbbVie: Grant/Research Support|Basilea: Grant/Research Support|Cipla: Grant/Research Support|Entasis: Grant/Research Support|GSK: Grant/Research Support|Paratek: Grant/Research Support|Pfizer: Grant/Research Support|Shionogi: Grant/Research Support

